# Zinc Binds to RRM2 Peptide of TDP-43

**DOI:** 10.3390/ijms21239080

**Published:** 2020-11-29

**Authors:** Andrey V. Golovin, Francois Devred, Dahbia Yatoui, Andrei Yu. Roman, Arthur O. Zalevsky, Remy Puppo, Regine Lebrun, Francoise Guerlesquin, Philipp O. Tsvetkov

**Affiliations:** 1 Faculty of Bioengineering and Bioinformatics, Lomonosov Moscow State University, Leninskie Gory 1, 119234 Moscow, Russian; golovin@fbb.msu.ru (A.V.G.); aozalevsky@fbb.msu.ru (A.O.Z.); 2 Sirius University of Science and Technology, 354340 Sochi, Russian; 3 Faculty of Computer Science, National Research University Higher School of Economics, 101000 Moscow, Russian; 4 Inst Neurophysiopathol, CNRS, INP, Aix Marseille Université, 13385 Marseille, France; francois.devred@univ-amu.fr (F.D.); yatouidahbia19@gmail.com (D.Y.); aroman279@yandex.ru (A.Y.R.); 5 Institute of Physiologically Active Compounds, RAS, 142432 Chernogolovka, Russian; 6 Plate-forme Protéomique, Institut de Microbiologie de la Méditerranée (IMM), CNRS FR 3479, Aix-Marseille Université, Marseille Protéomique (MaP), 13009 Marseille, France; rpuppo@imm.cnrs.fr (R.P.); rlebrun@imm.cnrs.fr (R.L.); 7 Laboratoire d’Ingénierie des Systèmes Macromoléculaires, UMR 7255, Institut de Microbiologie de la Méditerranée, CNRS, Aix-Marseille Université, 13009 Marseille, France; guerlesq@imm.cnrs.fr

**Keywords:** TDP-43, zinc, QM/MM

## Abstract

Transactive response DNA and RNA binding protein 43 kDa (TDP-43) is a highly conserved heterogeneous nuclear ribonucleoprotein (hnRNP), which is involved in several steps of protein production including transcription and splicing. Its aggregates are frequently observed in motor neurons from amyotrophic lateral sclerosis patients and in the most common variant of frontotemporal lobar degeneration. Recently it was shown that TDP-43 is able to bind Zn^2+^ by its RRM domain. In this work, we have investigated Zn^2+^ binding to a short peptide 256–264 from C-terminus of RRM2 domain using isothermal titration calorimetry, electrospray ionization mass spectrometry, QM/MM simulations, and NMR spectroscopy. We have found that this peptide is able to bind zinc ions with a K_a_ equal to 1.6 × 10^5^ M^−1^. Our findings suggest the existence of a zinc binding site in the C-terminal region of RRM2 domain. Together with the existing structure of the RRM2 domain of TDP-43 we propose a model of its complex with Zn^2+^ which illustrates how zinc might regulate DNA/RNA binding.

## 1. Introduction

Together with calcium and magnesium, zinc is one of the key redox-inert cations implicated in biological processes. Zinc is often known as a structural cation for DNA binding proteins. Many zinc binding proteins share a common zinc finger motif which could be used for DNA, RNA and protein recognition [1]. Nevertheless, alternative zinc binding sites are well known, for example, amyloid β_6–14_ [2] or some EF-hand calcium-binding proteins [3,4]. Because such sites, which usually have lower affinity for zinc, are abundant in cells [5], their role in protein functions and pathological aggregation has been getting more attention lately.

It should be noted that zinc is not only an important regulator of many vital processes in cells but also involved in the pathogenesis of a number of neurodegenerative disorders including Alzheimer’s and Parkinson’s disease (AD, PD) [6]. If not a causative agent, at least it can bind to a number of prone-to-aggregate proteins implicated in ND (such as amyloid-β, tau, FUS/TLS, α-Synuclein, etc.) and often favor their aggregation [7]. For example, zinc was found to play an important role in amyloid-β structure, dynamics [8], and aggregation [9,10]. Moreover, it is present at high concentrations in amyloid plaques [11]. All these prone-to-aggregate proteins share two common features: they can bind DNA and zinc has been reported as an inducer of their aggregation.

TDP-43 is a DNA and RNA-binding protein that regulates expression of coding RNA and long non-coding RNA (lncRNA) expression and transcription, splicing, stability, and transport of mRNA, thus playing a role in all steps of mRNA life cycle (see [12] for review). It is also associated with several neurodegenerative diseases (ND), including amyotrophic lateral sclerosis (ALS) [13] and frontotemporal lobar dementia (FTLD) [14,15]. It is present in the brains of victims in the form of intracellular tau-negative but ubiquitin-positive inclusions [15,16]. Moreover, TDP-43 containing inclusions are also detected in up to 57% of Alzheimer’s disease (AD) cases [17]. Even though zinc was reported as an inductor of the aggregation of endogenous TDP-43 in cells [18], originally, DNA/RNA-binding protein TDP-43 was not recognized as a zinc-binding protein. Yet, recently we have demonstrated that TDP-43 is able to bind Zn^2+^ by its RRM domain [19]. This binding significantly decreases RRM stability at physiological temperatures and thus could have an impact on TDP-43 functioning.

**Figure 1:**
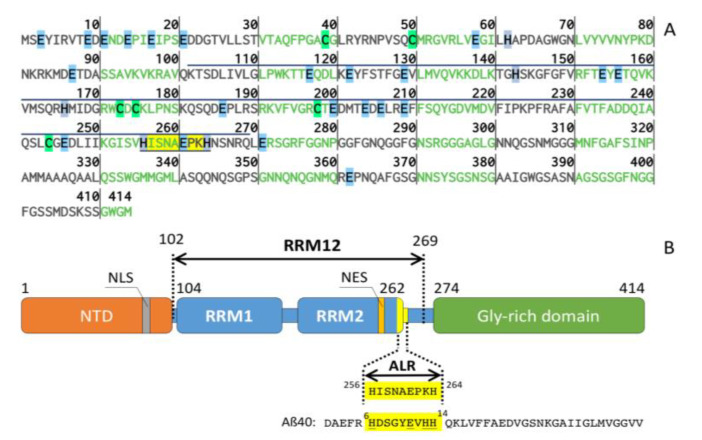
(**A**) Primary sequence of TAR DNA-binding protein 43 (TDP-43). The fragments of 102–269 (RRMs) and 256–264 (ALR) discussed in this study overlined and underlined respectively. The amino acids, which are able to chelate Zn^2+^ ions are highlighted: in clear blue—Glu, in green—Cys and in grey—His. (**B**) Domain organization of TDP-43: NTD—N-terminal domain; NLS—nuclear localization sequence; RRM1 and RRM2—RNA-recognition domains; NES—nuclear export signal. ALR in TDP-43 and Aβ_40_ sequence highlighted in yellow, amino acids that chelate Zn^2+^ in Aβ_40_ are underlined.

RRM domain of TDP-43, consists of two RNA recognition motifs RRM1 (104–200 a.a.) and RRM2 (201–262 a.a.) (Figure 1). Recently, it was suggested that conformational changes involving RNA recognition motifs RRM2 play a role in the aggregation and toxicity of the protein [20]. Close examination of RRM2 domain sequence revealed that sequence at C-terminus of RRM2 domain ^256^HISNAEPKH^264^ shares similarities with minimal zinc-binding site of amyloid-β (Aβ_6–14_) ^6^HDSGYEVHH^14^ (Figure 1), which is involved in zinc-induced dimerization of amyloid-β peptide [21]. Remarkably, this region was found partially unstructured in the NMR structure of the RRM complex with RNA [16]. This allowed us to hypothesize that this sequence of RRM domain could be directly involved in zinc binding. Thus, in this work, we studied zinc binding to ^256^HISNAEPKH^264^ peptide (called further ALR) from TDP-43, using isothermal titration calorimetry (ITC), mass-spectrometry (MS), and nuclear magnetic resonance (NMR). We also used quantum mechanics/molecular mechanics (QM/MM) methods to model the zinc-peptide complex formed.

## 2. Result and Discussion

### 2.1. Zinc Binding to a Peptide from TDP-43 Is Enthalpy Driven

**Figure 2:**
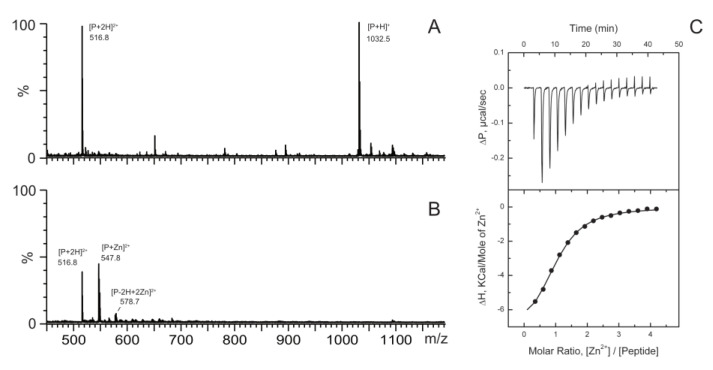
The 450–1200 *m*/*z* range of the positive ion mass spectra obtained for 10 µM ALR peptide in 100 mM ammonium acetate buffer in the absence (**A**) in the presence (**B**) of 50 mM ZnCl_2_, pH 7.3. Major signals at *m*/*z* 516.8, and 1032.5 correspond to the single and double charged molecular ion of the ALR peptide (marked as P). In the presence of zinc signal at 547.8 and 578.7 *m*/*z* were detected, which correspond to ALR complexes with one and two zinc ions. (**C**) Typical ITC titration curve (upper panel) and binding isotherm (lower panel) for zinc interactions with ALR peptide at 25 °C in 50 mM Tris buffer, pH 7.3.

Sequence similarity between Aβ_1–16_ and ^256^HISNAEPKH^264^ peptide (ALR) from TDP-43 provided motivation to tests that zinc binds to the C-terminus of RRM2. We investigated complex formation between zinc ions and ALR peptide form TDP-43 (Figure 1). We have used electrospray ionization mass spectrometry (ESI-MS), which allowed non-covalently bound multimeric peptide species to be detected [22,23]. All samples containing 10 µM of peptide were prepared in 100 mM ammonium acetate buffer at pH 7.5 without zinc or with 50 µM of ZnCl_2_. The mass spectrum of zinc-free peptide solution shows only intense signals that correspond to mono- and di-protonated ALR monomers at 1032.5 and 516.7 *m*/*z* respectively (Figure 2A). In the presence of zinc ions, we observed the formation 1:1 complex between ALR peptide and zinc ion (Figure 2B). No peaks corresponding to ALR dimer or higher-order oligomers were observed both in the absence and in the presence of zinc ions. It should be noted that in the presence of 5-fold excess of zinc ions, the peak at 578.7 Da, which corresponds to di-deprotonated peptide species bound with two zinc ions, was also observed but was minor in comparison with both free and single zinc-bound peptide species.

Thermodynamic characterization of zinc/peptide binding was performed using ITC (Figure 2C). Titration experiments revealed that ALR peptide binds zinc ions with an association constant of 1.6 ± 0.3 × 10^5^ M^−1^. This constant seems relatively low in comparison with free zinc concentration in cells. Yet it is similar to the one of the minimal zinc-binding site of Aβ (0.9 ± 0.3 × 10^5^ M^−1^ for Aβ_6–14_ [2]) which is widely accepted to play an important role in Aβ pathological aggregation [24]. Indeed, in pathological conditions, oxidation of cysteines, major zinc chelating amino acids, can release zinc from metalloproteins resulting in a strong increase in local zinc concentration in neurons [3,25].

The enthalpy of zinc interaction with Aβ_6–14_ and ALR peptides were also close to each other (−9.2 kcal/mol [2] and −7.6 kcal/mol respectively) showing that both processes are enthalpy driven. Meanwhile, zinc binding to ALR is an entropy unfavorable process (−1.6 cal/K mol), while its binding to Aβ_6–14_ strongly entropy favorable (8.3 cal/K mol) [2]. This indicates that contrary to Aβ_6–14_ there is no entropically favorable burying of hydrophobic contacts upon zinc interaction with ALR, the basis of this observation probably lies in the fact that ALR peptide has only Ile hydrophobic residue than Aβ_6–14_ has Tyr and Val.

### 2.2. NMR Analysis of Zinc Coordination in ALR Peptide Points to Multiple Conformation Complex Supports

**Figure 3:**
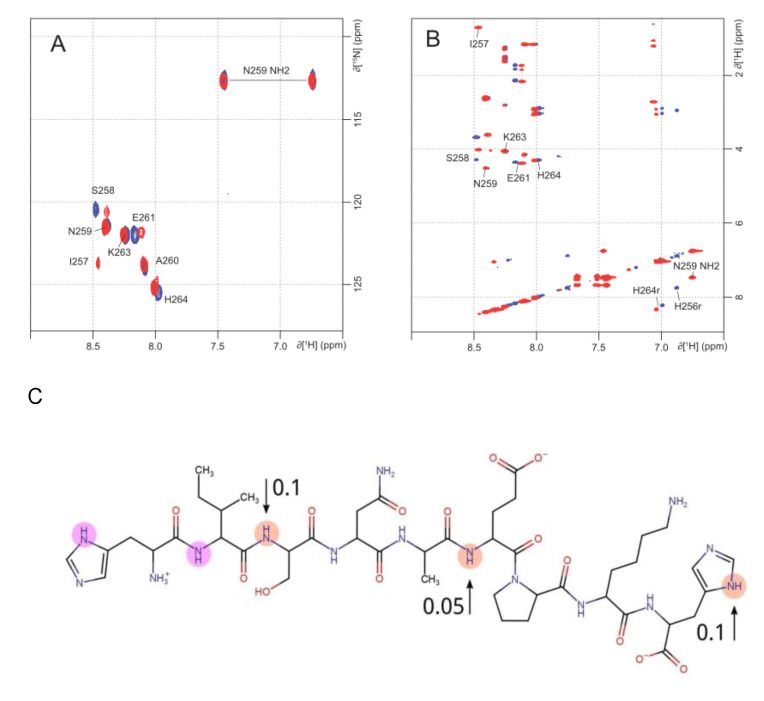
(**A**) 1H-15N HSQC spectra of ALR peptide (in blue) recorded at natural abundance for a sample at 0.8 mM concentration, 290 K and in phosphate buffer pH 6.8. For Zn^2+^/ALR complex (in red) the same conditions were used with a ratio of 10/1. NH group and Asn259 side chain NH2 assignments are indicated. (**B**) Zoom in on the homonuclear TOCSY spectra of free (in blue) and Zn^2+^ bound (in red) ALR peptides. (**C**) Chemical structure of ALR peptide. Atoms highlighted with red circles correspond to atoms with chemical shift upon zinc binding greater than 0.05. Atoms highlighted with pink circles correspond to atoms with new peaks upon zinc binding. The NMR spectra were recorded on a Bruker 600 MHz (Billerica, MA, USA) equipped with a TCI cryoprobe at 290 K. ALR peptide was at 0.8 mM concentration in a 10 mM phosphate buffer at pH 6.8. The spectra of Zn^2+^/ALR complex were obtained at a ratio 10:1. Sequential assignments of NH groups and side-chain protons of the peptide are indicated using protein numbering.

Zinc coordination in the fragment ALR was investigated by NMR. Proton assignments of free and Zn^2+^ bound ALR peptide were determined using TOCSY and NOESY homonuclear experiments. The NMR assignments are presented in Table 1. The presence of Zn^2+^ induced some significant chemical shift changes on NH groups as illustrated by ^1^H-^15^N HSQC (Figure 3A). Ser258 and His264 are found notably affected. This result is in perfect agreement with their expected role of Zn ligand. The third one His256 was not observable in this spectrum without Zn^2+^ because of the flexibility of the N-terminal extremity of the peptide. It is to be noticed that in the presence of Zn^2+^, Ile257, which was not observable in the free peptide spectra, appeared. These exchange modifications within the peptide spectra are probably due to a stabilization of the N-terminal extremity in the presence of Zn^2+^. Finally, NH group from the backbone of Glu261 was also found highly affected by the presence of Zn^2+^ suggesting that this residue may be the fourth ligand. Zn^2+^ NMR titration experiments were carried out on ALR peptide using TOCSY and NOESY homonuclear experiments. As described in ^1^H-^15^N HSQC, one can observe that increased concentrations of zinc affect the resonances of the NH groups of Ser258, Glu261, and His264. Moreover, the ring protons of His256 and His264 were also affected by the presence of Zn (Figure 3B).

Unfortunately, an unambiguous determination of the residue participation in zinc coordination due to NH peak displacement is impossible. Previously, it was shown [26] that in amyloid peptide Aβ_40_ a significant part of residues has the NH peak displacement due to zinc binding. This is caused by peptide-peptide interactions and the formation of secondary structure elements. Thus, in our case, most probably the NH peak displacement is caused by the participation of oxygen atoms from the peptide bond in zinc coordination. So, the number of potential chelators exceeds 4, which may indicate the presence of different conformations of the complex.

### 2.3. Comparative Simulations of All the Available Ways to Bind Zinc in ALR Peptide

NMR data allow unambiguously to identify two histidine residues His256 and His264 as zinc chelators in ALR/Zinc complex. Unfortunately, such clear identification of other zinc chelators is impossible with our NMR data. Earlier, we successfully used QM/MM modeling for studying the structure and dynamics of zinc ions complexes with short peptides from amyloid β [22]. Here we used a similar approach to identify two missing chelators of zinc which led to the formation of the most stable complex of ALR/zinc.

Excluding histidine residues, there are 12 oxygen atoms in ALR that have the potential to act as zinc chelators, namely: two Oε oxygens of Glu261 side chain, Oδ oxygen of Asn259, Oγ oxygen of Ser258, and 8 backbone oxygens. Here and later, we are using common IUPAC-IUB nomenclature for amino acids atoms names. Analysis of existing PDB structures revealed a very low amount of complexes (24 from 18,491 [27] or 12 from 6150 for resolution less than 2 Å [3]) wherein zinc ion is chelated by asparagine and serine residues, while glutamate is one of the most common chelators of zinc after cysteine and histidine. Thus, we created 10 initial complexes of ALR peptide with zinc wherein zinc is chelated with two histidine residues His256 and His264, Oε oxygen of Glu261 side chain, and one of 10 other possible oxygen atoms.

These complexes have been constructed on the basis of the ideal geometry of four-way zinc coordination described with the force field of parm99 [28]. The geometry of each system was optimized and balanced in an explicitly defined solvent when modeling molecular dynamics with a 200 ns long trajectory. After optimization, all complexes came to a stable geometry in terms of RMSD (RMSD was lower than 2.5 Å in the last 50 ns of the trajectory) of the whole peptide wherein zinc coordination was maintained by means of distance and angular restraints for zinc chelating atoms.

Then to estimate zinc-binding, energy simulation of QM/MM metadynamics was used. MM subsystem was described with force field parm99SB with corrections [29], while simulations of the QM subsystem was described with the DFTb/3OB potential. The QM subsystem included sidechain radicals and backbone atoms of residues which had oxygen atom at the distance within 6 Å from zinc ion. Water molecules were included in the QM subsystem by the same rule. We used a collective variable [30] describing the coordination of zinc by any nitrogen and oxygen atoms. Modeling of metadynamics using such a collective variable allowed to estimate both the stability of the zinc coordination sphere and to observe the exchange of zinc chelators in the coordination sphere, including water. The exchange of chelators makes it difficult to determine the interaction energy of a peptide with zinc for a specific set of chelators. To circumvent this limitation, we have used a reweighting algorithm to reconstruct the free energy surface (FES) [31] for each combination of chelators. The re-weighing allowed estimating the energy of interaction for every four chelators on the basis of the already simulated metadynamics trajectories. Further, we considered only chelators that belong to peptide residues.

**Figure 4:**
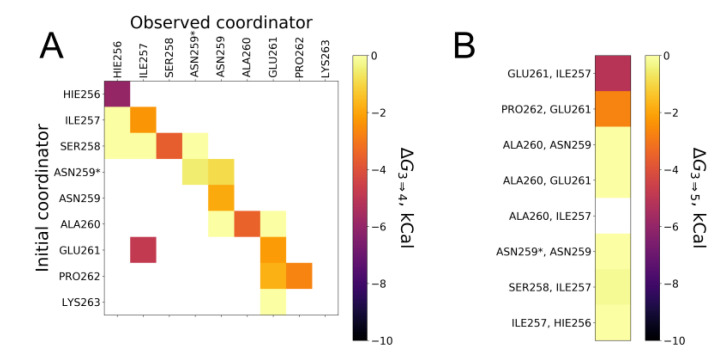
Screening for fourth and fifth chelators for Zn^2+^ in ALR peptide while other chelators are: Nδ His256, Nδ His264, and Oδ Glu261. All chelators’ names correspond to the backbone oxygen atom, except ASN259* is Oδ. ΔG values were estimated from simulations of systems initially built according to the abscissa names, while the axis represents observed coordination and corresponding energy values. (**A**) Matrix of ΔG values between four and three-coordinate state of Zn^2+^, (**B**) Column of ΔG values where the five-coordination sphere of Zn^2+^ was observed in simulation.

The result of the re-weighing is presented in Figure 4A as a matrix of values of free energy change (ΔG_3⇒4_). ΔG_3⇒4_ is the free energy change from three zinc chelators state to four zinc chelators. Only peptide chelators were taken into account. In case of the absence of chelators exchange in the coordination sphere during the re-weighing procedure, all values of binding energies lie on the matrix diagonals. The presence of the non-diagonal values different from zero for the three systems points to the chelators exchange or to the formation of five-coordinated complexes with zinc. Re-weighing the metadynamic data for the five-coordination state of zinc has shown that a stable five-coordination sphere is formed with the participation of two backbone oxygens from Glu261/Ile257 and Glu261/Pro262 (Figure 4B). In this case, the presented values of free energy variation were defined as differences between states with five and three chelators (ΔG_3⇒5_).

**Figure 5:**
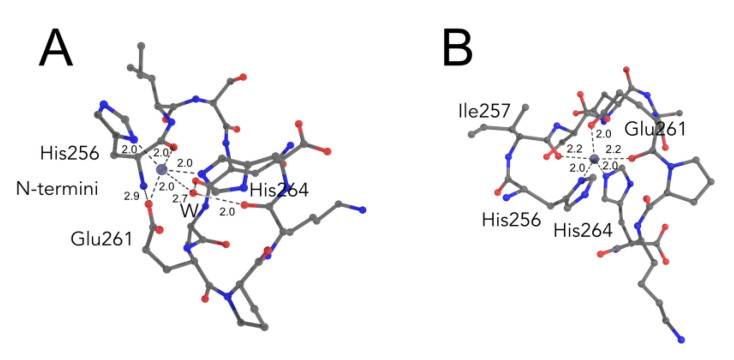
ALR peptide models from QM/MM simulations in which the zinc ion is coordinated (**A**) by four chelators: Nδ His256, Nδ His264, and Oδ Glu261 and backbone O from His256. (**B**) by five chelators: Nδ His256, Nδ His264, Oδ Glu261, backbone O from Glu261, and backbone O from Ile257. Oxygen atoms colored in red, nitrogen atoms in blue, carbon atoms in grey, and zinc cation in slate blue. Dashed lines present distances in the coordination sphere. Atom names are given according to the IUPAC-IUB convention.

The best energy (ΔG_3⇒5_) of −6.5 kcal/mol, was found for the system where the fourth zinc chelator is the backbone oxygen atom of His256. This value is close to the experimental value of the complex formation of −7.1 kcal/mol as measured by ITC. The structure of the complex is stabilized by the interaction of the charged N-terminal amino group with the carboxyl group of Glu261, thus positioning the backbone oxygen of His256 in the coordination sphere of zinc (Figure 5A). Zinc coordination by the backbone oxygen of His256 atom in the simulation is in line with the NMR data where a new peak of NH group from the peptide bond between His256 and Ile257 appeared upon the complex formation. The water molecule is effectively positioned by the backbone oxygen atoms of Asn255 and Lys263 and can serve as a supplementary chelating factor for zinc and another complex stabilizing factor (Figure 5A).

In the case of the most stable pentacoordinate system, zinc is coordinated by two backbone oxygen atoms from Glu261 and Ile257 (Figure 5B). The energy of transition to three-coordinated state (ΔG_3⇒5_) for this system is equal to −5.5 kcal/mol, which differs from the experimentally found value of −7.1 kcal/mol. Zinc coordination by the Ile257 and Glu261 oxygen atoms in the simulation also correlates with the NMR data, where a shift of the peak of backbone NH group of Ser258 on 1H-15N HSQC spectra was observed upon zinc chelation. Indeed, such a shift might be caused by zinc coordination by the oxygen atom from the peptide bond, which belongs to this backbone NH group, i.e., the Ile257 residual backbone oxygen. Unfortunately, we could not observe the peak shift from the amino group of the peptide bond Glu261-Pro262 due to the absence of backbone NH proton in the proline residue. Nevertheless, we observed an insignificant shift in the backbone NH of the Glu261 group.

Thus, the two described coordination modes of zinc by ALR peptide fully explain the shift of peaks of backbone NH-groups in NMR spectra. To extrapolate obtained data on zinc coordination in the RRM domain of TDP-43 it is necessary to take into account the absence of charged amino group residue of His256, then the second mode of coordination is more preferable in TDP-43. The involvement of His264 and Glu261 in zinc coordination can significantly change the conformation of Lys263, which is a target for ubiquitin ligation.

### 2.4. Biological Relevance

In summary, we found that zinc binds to peptide 256–264 from the C-terminal region of the RRM2 domain of TDP-43 and induces its structurization. Since this 256–264 region has been shown to be partially unfolded within a RRM1-RMM2 construct [32], we hypothesize that this change in conformation may be relevant in the context of the entire protein. Indeed, zinc has been shown to play a central role in the structuration of a wide variety of proteins [1] even including intrinsically disordered proteins (IDPs) such as amyloid-β [2,26], tau [33], Prothymosin α [34,35] or Human brain derived neurotrophic factor (BDNF) [36].

Structuration of this region of TDP-43 upon zinc binding could have consequences on the physiological role of TDP-43 and/or its implication in neurodegenerative processes. Indeed, taking into account the localization of studied peptide fragment in the protein, it is possible to hypothesize that, when such zinc interaction occurs in full-length TDP-43, it could impact its interaction with RNA. It should be noted that in comparison with zinc-finger proteins (ZFP) that also binds DNA and RNA, TDP-43 has a much lower constant. Thus, in contrast to ZFPs, wherein zinc plays mostly the role of structural cofactor, in TDP-43, zinc could play a regulatory role. To our best knowledge none of heterogeneous nuclear ribonucleoproteins were reported as zinc binding, except FUS protein, which contains, in addition to a RRM region, a zinc finger domain [37]. Interestingly, as TDP-43, FUS protein also aggregates in ALS.

Recently, zinc involvement in aggregation of another RNA-binding protein SFPQ, that also contains two RRM domains, was reported [38,39]. However in that case, the zinc binding site was not located in RRM domains, but in an interface formed by long α-helixes of four SFPQ subunits [38] revealing the potential molecular mechanism of SFPQ polymerization. Together with the previous results on zinc-induced aggregation of TDP-43 in cells [18] and in vitro [19] we hope that our results will contribute in elucidating the molecular mechanism of this process too and its implication in neurodegeneration.

## 3. Materials and Methods

### 3.1. Materials

Chemicals and solvents were of HPLC-grade or better and were obtained from Sigma-Aldrich (St. Louis, MO, USA). Synthetic peptides TDP-43_(256–264)_ named here ALR: HISNAEPKH (purity > 98%, checked by RP-HPLC) were purchased from GeneCust Europe. The solubility of the peptide was controlled spectrophotometrically. Peptide solutions were centrifuged before usage.

### 3.2. Mass-Spectrometry

Experiments were carried out on an electrospray Q-ToF mass spectrometer (Synapt G1, Waters). Soft ionization parameters were used to prevent in source dissociation of the zinc-peptide complexes. The following settings were used: spray voltage 3.2 kV, sampling cone 75, source temperature 110 °C, trap CE 4, transfer CE 4, and source vacuum increased to 4 mBar. Peptide solution (10 µM in ammonium acetate 100 mM) with or without 50 µM ZnCl_2_ was used for the experiments. Mono isotopic peaks were measured.

### 3.3. Isothermal Titration Calorimetry (ITC)

Thermodynamic parameters of zinc binding to ALR peptide were measured using a MicroCal ITC200 (Malvern, UK) as described previously [2]. Experiments were carried out in 50 mM Tris buffer, pH 7.3 at 25 °C. Aliquots of ZnCl_2_ solution (2 μL) were injected into the 0.2 mL cell containing a solution of peptide or protein to achieve a complete binding isotherm. Peptide and concentration in the cell ranged from 0.1 to 0.5 mM and the ZnCl_2_ concentration in the syringe ranged from 2 to 9 mM. The heat of dilution was measured by injecting the ligand (ZnCl_2_) into the buffer solution; the values obtained were subtracted from the heat of reaction to obtain the effective heat of binding. The resulting titration curves were fitted using Origin software (7.0, OriginLab, Northampton, MA, USA). The association constant (K_a_) and binding stoichiometry (N) were determined by a non-linear regression fitting procedure [4].

### 3.4. NMR Experiments

1D-NMR spectra were recorded at 290 K on a Bruker 600 MHz spectrometer (Billerica, MA, USA) equipped with a TCI cryoprobe, on a 0.8 mM ALR peptide sample in 10 mM phosphate buffer at pH 6.8, containing 10% D_2_O. Proton assignments were obtained by recording TOCSY and NOESY experiments (Table 1).^1^H-^15^N HSQC spectra were recorded at a natural abundance of ^15^N. NMR titration of Zinc binding to ALR peptide was obtained by following 1D proton, 2D NOESY, 2D TOCSY, and 2D ^1^H-^15^N HSQC spectra recorded at various ZnCl_2_ concentrations (ratio Zn/ALR: 0, 1, 2, 3, 4, 5, and 10).

### 3.5. QM/MM Modeling

The starting conformation of ALR peptide was extracted from NMR solution structure of RRM2 of TDP-43 (PDBID:1WF0). Distance from zinc to chelating atoms from residues His256(Nδ), His264(Nδ) and Glu261(Oδ1) was restrained with previously found parameters for the parm99 force field [40]. Classical simulations were performed with GROMACS package with a velocity thermostat with a stochastic component with constant volume. In QM/MM simulations, a molecular mechanics approach was applied to MM part of the system using the parameters from the parm99 force field with corrections by [29]. The QM subsystem was described with DFTB3-D3 Hamiltonian [29,41,42] with special parametrization of Zn^2+^ interactions [43]. Side-chain atoms of His256, His264, Glu261, and selected backbone atoms were included in the QM part of the system. Each simulation system was filled with water molecules presented by the tip3p model, and the total charge was neutralized with Na^+^ or Cl^−^ ions. Water and ions were equilibrated around the peptide-zinc complex by carrying out a 200 ns MD simulation with a restrained position of QM part of the system. Additionally, any atom including water closer to zinc than 3 Å and its surroundings were included in the QM part. The prepared systems were subjected to QM/MM simulation with GROMACS-DFTb/Plumed package [30,44]. The time step used was 0.2 fs. Temperature coupling with velocity rescale scheme with stochastic correction. The total length of the simulation was set to 5600 ps. To overcome the coordination energy barrier metadynamics approach was used. We used one collective variable: coordination number of Zn by all possible chelators in the QM subsystem. Several different metadynamics parameters (hill height, width, and pace) were tested, hill with sigma 0.02 and height 1 kJ/mol was chosen, hills pace was selected as one hill every 250th step. Final analysis of metadynamics data was done with a reweighting procedure to reconstruct the free energy surface (FES) for all combinations of chelators in all systems. From FES after reweighting procedure, energy values for 3,4,5-way zinc chelation by peptide were extracted by search of local minima. Energy change from state with 3 peptide chelators to state with 4 chelators was used for presentation. The same procedure was used for complexes with 5-way zinc coordination. Energy change from 3 zinc chelators state to 5 was used for presentation.

## 4. Conclusions

In conclusion, we investigated a short HISNAEPKH peptide that corresponds to the partially disordered region 256–264 of RRM2 domain of TDP-43 using ITC, MS, NMR, and QM/MM modeling and found that it can coordinate one zinc ion by Nδ His256, Nδ His264, and Oδ Glu261 and backbone O from His256 with a K_a_ equal to 1.6 × 10^5^ M^−1^. This allowed us to hypothesis the potential implication of zinc in functional regulation of TDP-43 and/or its pathological aggregation.

## Figures and Tables

**Table 1:** Proton chemical shifts of ALR peptide at 290 K and pH 6.8. NH indicates proton in NH groups, HA, the protons on the CA and Hsc, protons on the side chains.

#	A.A.	Zn^2+^	NH	HA	Hsc	Hsc	Hsc	Hsc	Hsc	Hsc
256	His	−		3.95	2.94				6.87	7.74
		+								
257	Ile	−								
		+	8.46	3.99	1.63	0.67				
258	Ser	−	8.48	4.25	3.64					
		+	8.38		3.57					
259	Asn	−	8.39	4.51	2.56				6.74	7.47
		+	8.39	4.51	2.56				6.74	7.47
260	Ala	−	8.1	4.1	1.13					
		+	8.1	4.1	1.13					
261	Glu	−	8.17	4.31	2.1	1.79	1.67			
		+	8.12	4.28	2.09	1.8	1.66			
263	Lys	−	8.23	4.02	2.77	1.55	1.49	1.25		
		+	8.23	4.02	2.77	1.55	1.49	1.25		
264	His	−	7.98	4.25	2.99	2.85			6.99	8.23
		+	8.01	4.28	3.02	2.88			7.04	8.34

## Abbreviations

TDP-43	transactive response DNA binding protein 43 kDa
RRM	RNA recognition motif
ALS	amyotrophic lateral sclerosis
FTLD	frontotemporal lobar degeneration
AD	Alzheimer’s disease
NTD	N-terminal domain of TDP-43
ITC	isothermal titration calorimetry
Aβ_1–16_	amyloid-β metal-binding domain 1–16
